# Herpes simplex virus type 1 epidemiology in the Middle East and North Africa: systematic review, meta-analyses, and meta-regressions

**DOI:** 10.1038/s41598-018-37833-8

**Published:** 2019-02-04

**Authors:** Sonia Chaabane, Manale Harfouche, Hiam Chemaitelly, Guido Schwarzer, Laith J. Abu-Raddad

**Affiliations:** 1 Infectious Disease Epidemiology Group, Weill Cornell Medicine-Qatar, Cornell University, Qatar Foundation - Education City, Doha, Qatar; 2 Institute of Medical Biometry and Statistics, Faculty of Medicine and Medical Center - University of Freiburg, Freiburg, Germany; 3 Department of Healthcare Policy & Ressearch, Weill Cornell Medicine, Cornell University, New York, USA; 4 College of Health and Life Sciences, Hamad bin Khalifa University, Doha, Qatar

## Abstract

This study aimed at characterizing herpes simplex virus type 1 (HSV-1) epidemiology in the Middle East and North Africa (MENA). HSV-1 records were systematically reviewed. Findings were reported following the PRISMA guidelines. Random-effects meta-analyses were implemented to estimate pooled mean HSV-1 seroprevalence. Random-effects meta-regressions were conducted to identify predictors of higher seroprevalence. Thirty-nine overall seroprevalence measures yielding 85 stratified measures were identified and included in the analyses. Pooled mean seroprevalence was 65.2% (95% CI: 53.6–76.1%) in children, and 91.5% (95% CI: 89.4–93.5%) in adults. By age group, seroprevalence was lowest at 60.5% (95% CI: 48.1–72.3%) in <10 years old, followed by 85.6% (95% CI: 80.5–90.1%) in 10–19 years old, 90.7% (95% CI: 84.7–95.5%) in 20–29 years old, and 94.3% (95% CI: 89.5–97.9%) in ≥30 years old. Age was the strongest predictor of seroprevalence explaining 44.3% of the variation. Assay type, sex, population type, year of data collection, year of publication, sample size, and sampling method were not significantly associated with seroprevalence. The a *priori* considered factors explained 48.6% of the variation in seroprevalence. HSV-1 seroprevalence persists at high levels in MENA with most infections acquired in childhood. There is no evidence for declines in seroprevalence despite improving socio-economic conditions.

## Introduction

Herpes simplex virus type 1 (HSV-1) is a widespread and incurable infection^1,2^. Although this infection is usually asymptomatic^3^, the virus is shed frequently and subclinically^4,5^. Clinically-apparent HSV-1 infection most often manifests as orolabial herpes lesions^6,7^, but the virus causes a diverse spectrum of diseases including neonatal herpes, corneal blindness, herpetic whitlow, meningitis, encephalitis, and genital herpes^7,8^. The infection’s clinical manifestations depend on the virus’ initial acquisition portal^6,7^—oral-to-oral transmission leads to an oral infection^6,7^, and oral-to-genital transmission (through oral sex) leads to a genital infection^6,9,10^.

HSV-1 is endemic globally as indicated by the high HSV-1 antibody prevalence (seroprevalence) across regions^2,11,12^. Although HSV-1 is typically acquired in childhood^8^, changes in hygiene and socio-economic conditions appear to have reduced exposure during childhood in Western^11,13–20^ and Asian countries^21^. A large fraction of youth in these countries reach sexual debut with no protective antibodies against HSV-1 infection, and thus at risk of acquiring the infection genitally^6,22^. A growing evidence indicates that HSV-1 is overtaking HSV-2 as the leading cause of first episode genital herpes in Western^6,22–26^ and (apparently) Asian countries^21^. The extent to which such a transition in HSV-1 epidemiology is occurring in other global regions remains unknown.

In this context, we aspired to determine HSV-1 seroprevalence levels in the Middle East and North Africa (MENA), and to characterize the extent to which HSV-1 is the etiological cause of clinically-diagnosed genital ulcer disease (GUD) and clinically-diagnosed genital herpes. These aims were addressed by: (1) systematically reviewing and synthesizing available data on HSV-1 seroprevalence and HSV-1 viral detection in GUD and genital herpes, (2) estimating the pooled mean HSV-1 seroprevalence in different populations and across ages, and (3) assessing the associations and predictors of higher seroprevalence and sources of between-study heterogeneity.

This study is part of a series of ongoing investigations meant to inform efforts by the World Health Organization (WHO) and global partners to characterize the regional and global infection and disease burden of HSV infections, accelerate HSV vaccine development^27,28^, and explore optimal strategies for HSV-1 control.

## Methods

The methodology used in this study follows and adapts that used in a systematic review of HSV-1 seroprevalence and HSV-1 viral detection in GUD and genital herpes in Asia^21^.

### Data sources and search strategy

The present systematic review was informed by the Cochrane Collaboration handbook^29^, and was reported following the Preferred Reporting Items for Systematic Reviews and Meta-analyses (PRISMA) guidelines^30^. The PRISMA checklist can be found in Supplementary Table S1.

A systematic literature search was conducted up to October 8, 2017, in PubMed and Embase. The search criteria included exploded MeSH/Emtree terms to cover all subheadings, with no language or time restrictions. Another search was conducted up to December 1, 2017 in national and regional databases including: Index Medicus for the Eastern Mediterranean Region, Iraqi Academic Scientific Journals Database, Scientific Information Database of Iran, and PakMediNet of Pakistan. Search strategies can be found in Supplementary Box S1.

The MENA region definition included 23 countries: Afghanistan, Algeria, Bahrain, Djibouti, Egypt, Iran, Iraq, Jordan, Kuwait, Lebanon, Libya, Morocco, Oman, Pakistan, Palestine, Qatar, Saudi Arabia, Somalia, Sudan, Syria, Tunisia, the United Arab Emirates (UAE), and Yemen.

### Study selection and inclusion and exclusion criteria

Search results were imported into Endnote, where duplicate records were removed. Titles and abstracts of remaining records were screened independently by SC, MH, and HC, for relevance. Full texts of records deemed relevant or potentially relevant were retrieved for further screening. Bibliographies of relevant records and reviews were also screened for possible missing publications.

The inclusion criteria included any record reporting an HSV-1 seroprevalence measure, based on primary data and type-specific diagnostic assay such as glycoprotein-G-based enzyme-linked immunosorbent assays (ELISA).

The inclusion criteria also included any record reporting a proportion of HSV-1 viral detection in clinically-diagnosed GUD or in clinically-diagnosed genital herpes. The minimum sample size of included studies was 10, regardless of the outcome measure.

The exclusion criteria included case reports, case series, reviews, editorials, letters to editors, commentaries, qualitative studies, and animal studies. HSV-1 seroprevalence measures reported in <3 months-old infants were excluded since they may reflect maternal antibodies.

In this work, a “record” refers to a document (a publication) reporting an outcome measure of interest, while a “study” refers to the details pertaining to a specific outcome measure. Accordingly, one record may contribute multiple studies, and multiple records of the same study are considered as duplicates and only included once.

### Data extraction and data synthesis

The extracted information included: author(s), publication title, year(s) of data collection, publication year, country of origin, country of survey, city, study site, study design, study sampling procedure, study population and its characteristics (e.g., sex and age), sample size, HSV-1 outcome measures, and diagnostic assay. Data were double extracted from relevant records by SC, MH, and HC.

Extracted outcome measures were based on their stratification in the original record. Stratifications of seroprevalence measures were considered using a pre-defined sequential order that prioritizes first population type, followed by age bracket, and then age group. Age bracket included children (<15 years of age) and adults (≥15 years of age). Age groups included <10, 10–19, 20–29, and ≥30 years of age—a stratification informed by the actual available data of age-strata.

The extracted seroprevalence data were synthesized by population type according to the following definitions:

1. Healthy general populations encompassing groups of presumably healthy persons (for example, pregnant women or blood donors) and outpatients attending a healthcare facility for an inconsequential health condition.
2. Clinical populations encompassing any population with a serious clinical condition, or with a condition potentially related to a clinical manifestation of HSV-1 infection.
3. Other populations encompassing populations not fitting the above definitions, or populations with an unclear risk of having acquired HSV-1, such as sex workers and mixed health-status populations.

### Meta-analyses

Random-effects meta-analyses were conducted to estimate the pooled mean HSV-1 seroprevalence in MENA by population type, age bracket, and age group. Pooled means were calculated using DerSimonian-Laird random-effects models^31^ whenever ≥3 measures were available. The variance of the seroprevalence measures was stabilized using the Freeman-Tukey type arcsine square-root transformation^32^.

Cochran’s Q statistic was calculated to test for heterogeneity in the pooled seroprevalence measures^33,34^. I^2^ measure was calculated to assess the magnitude of between-study variation that is due to true variation in seroprevalence across studies rather than chance^33^. Prediction interval was estimated to characterize the heterogeneity in the seroprevalence measures^33^.

Sensitivity analyses were conducted using generalized linear mixed models (GLMM)^35^. The results were used to confirm the pooled mean HSV-1 seroprevalence estimates generated based on the Freeman-Tukey type arcsine square-root transformation, given a recently-identified potential pathology in this transformation^35^.

Meta-analyses were performed in R version 3.4.1^36^ using the meta package^37^.

### Meta-regressions

Univariable and multivariable random-effects meta-regression analyses, using log-transformed proportions, were conducted to identify associations and predictors of higher HSV-1 seroprevalence and sources of between-study heterogeneity. Associations were described using relative risks (*RR*s), 95% confidence intervals (CIs), and p-values.

Potential predictors were specified *a priori* and included: age bracket, age group, assay type, country’s income, population type, sample size (<100 versus ≥100), sampling method (probability-based sampling versus non-probability-based sampling), year of data collection, and year of publication. Factors with p-value < 0.1 in univariable analysis were eligible for inclusion in the multivariable model. Factors with p-value < 0.05 in the multivariable analysis were considered as statistically significant predictors.

Assay type consisted of five assay types for which data were available: ELISA, enzyme immunoassay (EIA), immunofluorescence assay (IFA), neutralizing antibody assay (Nab), and western blot. Of note, different assays used different cut-off points. For example, for HerpeSelect® 1 ELISA, sera with optical density index values ≥ 1.10 were considered seropositive and <0.90 seronegative, with the rest deemed equivocal^38,39^. Meanwhile, for Euroimmun Anti-HSV-1 ELISA, sera with optical density index values ≥ 1.10 were considered seropositive and <0.80 seronegative, with the rest deemed equivocal^39,40^.

Country’s income was determined based on the World Bank classification^41^ for the countries for which HSV-1 seroprevalence data were available: lower-middle-income countries (Egypt, Jordan, Morocco, Pakistan, Palestine, Sudan, Syria, and Yemen), upper-middle-income countries (Algeria, Iran, Iraq, and Lebanon), high-income countries (Qatar and Saudi Arabia), and mixed for samples including specimens from different countries.

Missing values in the year of data collection variable were imputed using the median of the values generated (for studies with data) for the difference between the year of data collection and the year of publication.

Meta-regressions were conducted in Stata/SE version 13^42^ using the package metareg^43^.

### Quality assessment

There are documented issues with the sensitivity and specificity of HSV-1 diagnostic methods^44,45^. Therefore, an expert advisor, Professor Rhoda Ashley Morrow from the University of Washington, was consulted and assessed the quality of each diagnostic method in each identified relevant study. Only studies with sufficiently reliable and valid assays were included. Further quality assessment of included studies was conducted as informed by the Cochrane approach for risk of bias (ROB)^29^ and precision assessment.

Studies’ assessment into *low* versus *high* ROB was based on two quality domains: sampling methodology (probability-based versus non-probability-based sampling), and response rate (≥80% versus <80%). For instance, if probability-based sampling was used in a given study, the study was classified with a *low* ROB for that domain. Studies with missing information for any of the domains were classified as having *unclear* ROB for that specific domain.

Studies were considered as having *high* (versus *low*) precision if the number of HSV-1 tested individuals was at least 100 participants. For an HSV-1 seroprevalence of 80% and a sample size of 100, the 95% CI is 70.8–87.3%—a reasonable 95% CI estimate for an HSV-1 seroprevalence measure.

## Results

### Search results and scope of evidence

**Figure 1:**
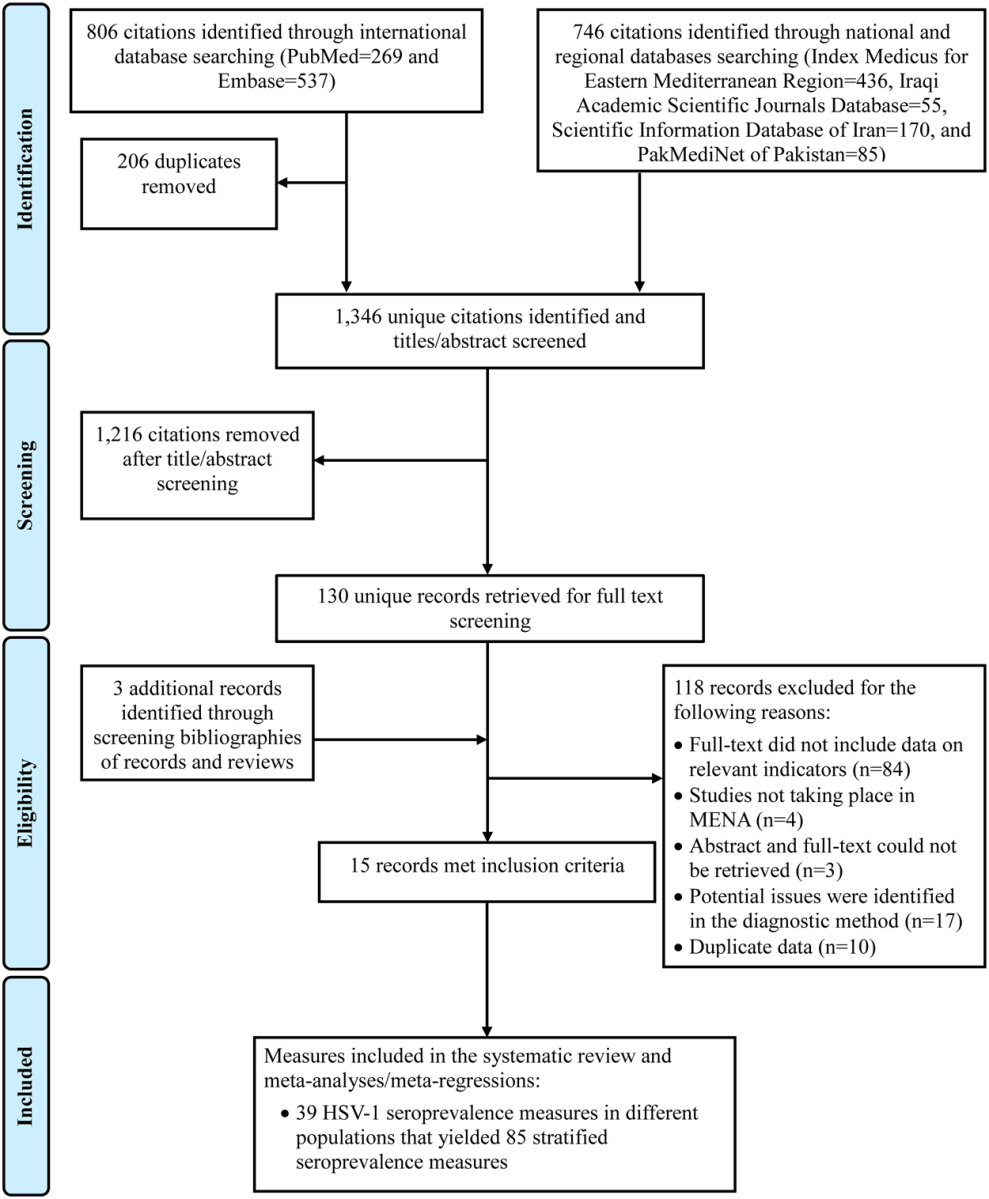
Flow chart of article selection for the systematic review of herpes simplex virus type 1 (HSV-1) in the Middle East and North Africa, as adapted from the PRISMA 2009 guidelines^30^. Abbreviations: HSV-1 = Herpes simplex virus type 1, MENA = Middle East and North Africa.

Figure 1 shows the process of study selection based on the PRISMA guidelines^30^. A total of 1,552 citations were retrieved (269 through PubMed, 537 through Embase, and 746 through national and regional databases). After duplicates’ removal and titles’ and abstracts’ screening, 130 records were identified as relevant or potentially relevant. Three additional records were identified through screening the bibliography of a previously published review for Iran^46^.

After full text screening, 15 records reporting an HSV-1 seroprevalence in 14 out of the 23 MENA countries were deemed relevant. Thirty-nine HSV-1 seroprevalence measures were extracted yielding 85 stratified measures. No HSV-1 seroprevalence measures (fulfilling the inclusion criteria) were identified among clinical children populations.

Although we searched for records that reported the proportion of GUD or genital herpes attributable to HSV-1, no such records were identified.

### HSV-1 seroprevalence overview

Table 1 summarizes the included HSV-1 seroprevalence measures. Studies included were published starting the year 1986, with the majority being cross-sectional in design and based on convenience sampling methods.

**Table 1:** Studies reporting herpes simplex virus type 1 (HSV-1) seroprevalence in the Middle East and North Africa.

Author, year	Year(s) of data collection	Country	Study site	Study design	Sampling method	Population	HSV-1 serological assay	Sample size	HSV-1 seroprevalence (%)
**Healthy children populations (n** = **21)**									
Cowan, 2003^53^	1998–00	Morocco	Outpatient clinic	CS	Conv	1–4 years old children	ELISA	159^b^	55.2
Cowan, 2003^53^	1998–00	Morocco	Outpatient clinic	CS	Conv	5–9 years old children	ELISA	159^b^	80.5
Cowan, 2003^53^	1998–00	Morocco	Outpatient clinic	CS	Conv	10–14 years old children	ELISA	160^b^	86.4
Ibrahim, 2000^54^	1995–98	Syria	Outpatient clinic	CS	Conv	1–5 years old males	ELISA	55	60.0
Ibrahim, 2000^54^	1995–98	Syria	Outpatient clinic	CS	Conv	6–10 years old males	ELISA	59	74.5
Ibrahim, 2000^54^	1995–98	Syria	Outpatient clinic	CS	Conv	1–5 years old females	ELISA	46	50.0
Ibrahim, 2000^54^	1995–98	Syria	Outpatient clinic	CS	Conv	6–10 years old females	ELISA	58	81.1
Meguenni, 1989^55^	—	Algeria	Community	CS	Conv	6 months-2 years old infants	Nab	34	23.5
Meguenni, 1989^55^	—	Algeria	Community	CS	Conv	3–5 years old children	Nab	33	39.4
Meguenni, 1989^55^	—	Algeria	Community	CS	Conv	6–10 years old children	Nab	36	69.4
Meguenni, 1989^55^	—	Algeria	Community	CS	Conv	11–15 years old children	Nab	32	81.3
**Healthy adult populations (n = 60)**									
Ahmed, 1995^56^	—	Pakistan	Outpatient clinic	CS^a^	Conv	Healthy controls	EIA	56	73.2
Hossain, 1986^57^	—	KSA	Outpatient clinic	CS	Conv	Pregnant women	IFA	1,186	92.0
Ibrahim, 2000^54^	1995–98	Syria	Outpatient clinic	CS	Conv	21–30 years old males	ELISA	48	85.4
Ibrahim, 2000^54^	1995–98	Syria	Outpatient clinic	CS	Conv	>30 years old males	ELISA	86	94.1
Ibrahim, 2000^54^	1995–98	Syria	Outpatient clinic	CS	Conv	21–30 years old females	ELISA	68	88.2
Ibrahim, 2000^54^	1995–98	Syria	Outpatient clinic	CS	Conv	>30 years old females	ELISA	43	95.3
Ibrahim, 2000^54^	1995–98	Syria	Outpatient clinic	CS	Conv	Pregnant women	ELISA	55	100
Jafarzadeh, 2011^58^	2007–08	Iran	Hospital	CS	Conv	>40 years old blood donors	ELISA	60	33.3
Meguenni, 1989^55^	—	Algeria	Community	CS	Conv	16–20 years old adults	Nab	32	87.5
Meguenni, 1989^55^	—	Algeria	Community	CS	Conv	21–30 years old adults	Nab	32	96.9
Meguenni, 1989^55^	—	Algeria	Community	CS	Conv	31–40 years old adults	Nab	30	100
Meguenni, 1989^55^	—	Algeria	Community	CS	Conv	>40 years old adults	Nab	35	100
Memish, 2015^59^	2012–13	KSA	Outpatient clinic	CS	RS	Healthy females	ELISA	2,157	90.9
Memish, 2015^59^	2012–13	KSA	Outpatient clinic	CS	RS	Healthy males	ELISA	2,828	87.1
Nabipour, 2006^60^	2004-04	Iran	Community	CS	Cluster RS	Healthy males	ELISA	881	83.8
Nabipour, 2006^60^	2003–04	Iran	Community	CS	Cluster RS	Healthy female	ELISA	910	88.6
Nasrallah, 2018^47^	2013–16	Mixed	Outpatient clinic	CS	Conv	Female blood donors	ELISA	88	84.1
Nasrallah, 2018^47^	2013–16	Pakistan	Outpatient clinic	CS	RS	Blood donor Pakistani males	ELISA	200	77.0
Nasrallah, 2018^47^	2013–16	Iran	Outpatient clinic	CS	Conv	Blood donor Iranian males	ELISA	113	81.4
Nasrallah, 2018^47^	2013–16	Sudan	Outpatient clinic	CS	Conv	Blood donor Sudanese males	ELISA	129	90.7
Nasrallah, 2018^47^	2013–16	Yemen	Outpatient clinic	CS	Conv	Blood donor Yemeni males	ELISA	148	92.6
Nasrallah, 2018^47^	2013–16	Egypt	Outpatient clinic	CS	Conv	≤24 years old blood donor Egyptians males	ELISA	50	92.0
Nasrallah, 2018^47^	2013–16	Egypt	Outpatient clinic	CS	Conv	25–29 years old blood donor Egyptians males	ELISA	50	100
Nasrallah, 2018^47^	2013–16	Egypt	Outpatient clinic	CS	Conv	30–34 years old blood donor Egyptians males	ELISA	50	98.0
Nasrallah, 2018^47^	2013–16	Egypt	Outpatient clinic	CS	Conv	35–39 years old blood donor Egyptians males	ELISA	50	98.0
Nasrallah, 2018^47^	2013–16	Egypt	Outpatient clinic	CS	Conv	40–44 years old blood donor Egyptians males	ELISA	50	98.0
Nasrallah, 2018^47^	2013–16	Egypt	Outpatient clinic	CS	Conv	45–49 years old blood donor Egyptians males	ELISA	50	100
Nasrallah, 2018^47^	2013–16	Egypt	Outpatient clinic	CS	Conv	50–54 years old blood donor Egyptians males	ELISA	39	94.9
Nasrallah, 2018^47^	2013–16	Egypt	Outpatient clinic	CS	Conv	≥55 years old blood donor Egyptians males	ELISA	19	100
Nasrallah, 2018^47^	2013–16	Qatar	Outpatient clinic	CS	Conv	≤24 years old blood donor Qatari males	ELISA	50	70.0
Nasrallah, 2018^47^	2013–16	Qatar	Outpatient clinic	CS	Conv	25–29 years old blood donor Qatari males	ELISA	50	62.0
Nasrallah, 2018^47^	2013–16	Qatar	Outpatient clinic	CS	Conv	30–34 years old blood donor Qatari males	ELISA	50	80.0
Nasrallah, 2018^47^	2013–16	Qatar	Outpatient clinic	CS	Conv	35–39 years old blood donor Qatari males	ELISA	50	82.0
Nasrallah, 2018^47^	2013–16	Qatar	Outpatient clinic	CS	Conv	40–44 years old blood donor Qatari males	ELISA	50	84.0
Nasrallah, 2018^47^	2013–16	Qatar	Outpatient clinic	CS	Conv	45–49 years old blood donor Qatari males	ELISA	50	96.0
Nasrallah, 2018^47^	2013–16	Qatar	Outpatient clinic	CS	Conv	50–54 years old blood donor Qatari males	ELISA	50	92.0
Nasrallah, 2018^47^	2013–16	Qatar	Outpatient clinic	CS	Conv	≥55 years old blood donor Qatari males	ELISA	50	92.0
Nasrallah, 2018^47^	2013–16	Jordan	Outpatient clinic	CS	Conv	Blood donor Jordanian males	ELISA	200	86.5
Nasrallah, 2018^47^	2013–16	Palestine	Outpatient clinic	CS	Conv	Blood donor Palestinians males	ELISA	200	80.5
Nasrallah, 2018^47^	2013–16	Syria	Outpatient clinic	CS	Conv	Blood donor Syrian males	ELISA	200	88.5
Nasrallah, 2018^47^	2013–16	Lebanon	Outpatient clinic	CS	Conv	Blood donor Lebanese males	ELISA	118	81.4
Obeid, 2007^61^	2004–04	KSA	Hospital	CS	Conv	Pregnant women	ELISA	459	84.1
Patnaik, 2007^62^	—	Morocco	Hospital	CS	Conv	Pregnant women	WB	169	98.8
Pourmand, 2009^63^	—	Iran	Outpatient clinic	CS	Conv	Pregnant women	ELISA	65	55.4
Ziyaeyan, 2007^64^	—	Iran	Hospital	CS	Conv	16–20 years old pregnant women	Nab	104	83.6
Ziyaeyan, 2007^64^	—	Iran	Hospital	CS	Conv	21–25 years old pregnant women	Nab	125	94.4
Ziyaeyan, 2007^64^	—	Iran	Hospital	CS	Conv	26–30 years old pregnant women	Nab	113	90.3
Ziyaeyan, 2007^64^	—	Iran	Hospital	CS	Conv	31–35 years old pregnant women	Nab	44	95.4
Ziyaeyan, 2007^64^	—	Iran	Hospital	CS	Conv	36–40 years old pregnant women	Nab	14	100
**Healthy age-mixed populations (n = 5)**									
Ibrahim, 2000^54^	1995–98	Syria	Outpatient clinic	CS	Conv	11–20 years old males	ELISA	57	77.1
Ibrahim, 2000^54^	1995–98	Syria	Outpatient clinic	CS	Conv	11–20 years old females	ELISA	134	83.6
RezaeiC, 2012^65^	2010–11	Iran	Outpatient clinic	CS	Cluster RS	Healthy population	ELISA	800	58.4
RezaeiC, 2012^66^	2010–11	Iran	Outpatient clinic	CS	Cluster RS	<85 years old patients	ELISA	200	65.5
**Clinical adult populations (n = 20)**									
Ibrahim, 2000^54^	1995–98	Syria	Outpatient clinic	CS	Conv	Patients with labials herpes	ELISA	36	100
Ibrahim, 2000^54^	1995–98	Syria	Outpatient clinic	CS	Conv	Patients with atherosclerosis	ELISA	60	100
Ibrahim, 2000^54^	1995–98	Syria	Outpatient clinic	CS	Conv	Kidney transplant patients	ELISA	32	96.9
Ibrahim, 2000^54^	1995–98	Syria	Outpatient clinic	CS	Conv	Patients with herpetic keratitis	ELISA	14	85.7
Ibrahim, 2000^54^	1995–98	Syria	Outpatient clinic	CS	Conv	Patients with STDs	ELISA	21	90.5
Ibrahim, 2000^54^	1995–98	Syria	Outpatient clinic	CS	Conv	Patients with cervical cancer	ELISA	51	100
Ibrahim, 2000^54^	1995–98	Syria	Outpatient clinic	CS	Conv	HIV positive patients	ELISA	25	96.0
Jafarzadeh, 2011^58^	2007–08	Iran	Hospital	CS	Conv	Patients with myocardial infarction	ELISA	120	60.8
Janier, 1999^67^	1994–94	Mixed	Outpatient clinic	CS	Conv	Patients with STDs	EIA	99	98.9
**Clinical age-mixed population (n = 4)**									
Ibrahim, 2000^54^	1995–98	Syria	Outpatient clinic	CS	Conv	Patients with encephalitis	ELISA	51	58.8
Ibrahim, 2000^54^	1995–98	Syria	Outpatient clinic	CS	Conv	Patients with meningitis	ELISA	21	85.7
**Other populations (n = 10)**									
Cowan, 2003^53^	1998–00	Morocco	Outpatient clinic	CS	Conv	15–19 years old healthy and HIV infected adults	ELISA	494^b^	92.2
Cowan, 2003^53^	1998–00	Morocco	Outpatient clinic	CS	Conv	20–29 years old healthy and HIV infected adults	ELISA	494^b^	92.1
Cowan, 2003^53^	1998–00	Morocco	Outpatient clinic	CS	Conv	30–34 years old healthy and HIV infected adults	ELISA	494^b^	95.0
Cowan, 2003^53^	1998–00	Morocco	Outpatient clinic	CS	Conv	35–39 years old healthy and HIV infected adults	ELISA	494^b^	98.8
Cowan, 2003^53^	1998–00	Morocco	Outpatient clinic	CS	Conv	40–45 years old healthy and HIV infected adults	ELISA	494^b^	100
Cowan, 2003^53^	1998–00	Morocco	Outpatient clinic	CS	Conv	>45 years old healthy and HIV infected adults	ELISA	493^b^	100
Ibrahim, 2000^54^	1995–98	Syria	Community	CS	Conv	Female sex workers	ELISA	54	100
Ibrahim, 2000^54^	1995–99	Syria	Community	CS	Conv	Female sex workers	ELISA	47	100
Ibrahim, 2000^54^	1995–100	Syria	Community	CS	Conv	Arab bar-girls	ELISA	50	98.0
Ibrahim, 2000^54^	1995–101	Syria	Community	CS	Conv	Foreign bar-girls	ELISA	75	92.0

^a^Actual study design was cohort but the extracted seroprevalence measure was for the baseline measurement.

^b^Study included overall sample size, but no individual strata sample sizes. Each stratum sample size was assumed equal to overall sample size divided by the number of strata in the study.

Abbreviations: Conv = Convenience, CS = Cross-sectional, EIA = Enzyme immunoassay, ELISA = Enzyme-linked immunosorbent assay, HIV = Human immunodeficiency virus, HSV-1 = Herpes simplex virus type 1, IFA = Indirect fluorescent assay, KSA = Kingdom of Saudi Arabia, Nab = Neutralization test with neutralizing antibody, RS = Random sampling, STD = Sexually transmitted disease, TORCH = Toxoplasmosis, other (syphilis, varicella-zoster, parvovirus B19), rubella, cytomegalovirus, and herpes infections, WB = Western blot.

Stratified HSV-1 seroprevalence measures (number of studies (n) = 85) varied across studies and ranged between 23.5–100% with a median of 90.3% (Table 2). The 11 seroprevalence measures in healthy children populations ranged between 23.5–86.4% with a median of 69.4%. The 49 seroprevalence measures in healthy adult populations ranged between 33.3–100% with a median of 90.7%. The 9 seroprevalence measures in clinical adult populations ranged between 60.8–100% with a median of 96.9%.

**Table 2:** Pooled mean estimates for herpes simplex virus type 1 (HSV-1) seroprevalence in different populations in the Middle East and North Africa.

Population type	Studies	Samples	HSV-1 seroprevalence		Pooled mean HSV-1 seroprevalence	Heterogeneity measures		
	Total N	Total n	Range	Median	Mean (95% CI)	Q^a^ (p-value)	I²^b^ (%) (95% CI)	Prediction Interval^c^ (%)
**Healthy general populations**								
Children	11	831	23.5–86.4	69.4	65.2 (53.6–76.1)	109.2 (p < 0.0001)	90.8 (85.6–94.2)	22.3–97.1
Adults	49	11,754	33.3–100	90.7	89.4 (87.3–91.4)	429.6 (p < 0.0001)	88.8 (86.1–91.0)	74.3–98.6
Age-mixed	4	1,191	58.4–83.6	71.3	71.1 (58.5–82.3)	42.1 (p < 0.0001)	92.9 (85.0–96.6)	13.7–100
All healthy general populations	64	13,776	23.5–100	86.5	85.3 (82.3–87.9)	1,107.9 (p < 0.0001)	94.3 (93.2–95.1)	59.4–99.4
**Clinical populations**								
Children	—	—	—	—	—	—	—	—
Adults	9	458	60.8–100	96.9	95.3 (83.9–100)	114.4 (p < 0.0001)	93.0 (88.9–95.6)	38.1–100
Age-mixed	2	72	58.8–85.7	72.2	66.7 (54.6–77.3)	—	—	—
All clinical populations	11	530	58.8–100	96.0	92.3 (80.3–99.4)	147.1 (p < 0.0001)	93.2 (89.7–95.5)	33.7–100
**Other populations**								
Female sex workers	4	226	92.0–100	99.0	95.2 (75.4–100)	57.8 (p < 0.0001)	94.8 (89.7–97.4)	0.0–100
Healthy/clinical adult populations	6	2,963	92.1–100	96.9	97.5 (93.5–99.7)	151.4 (p < 0.0001)	96.7 (94.7–97.9)	74.8–100
**Age group**								
<10 years	9	639	23.5–81.1	60.0	60.5 (48.1–72.3)	73.6 (p < 0.0001)	89.1 (81.6–93.6)	18.4–95.0
10–19 years	7	1,013	77.1–92.2	83.6	85.6 (80.5–90.1)	20.5 (p = 0.0023)	70.7 (36.0–86.6)	68.5–96.9
20–29 years	8	980	62.0–100	91.2	90.7 (84.7–95.5)	43.8 (p < 0.0001)	84.0 (70.2–91.4)	66.2–100
≥30 years	24	2,965	33.3–100	95.7	94.3 (89.5–97.9)	433.2 (p < 0.001)	94.7 (93.2–95.9)	60.9–100
All children	11	831	23.5–86.4	69.4	65.2 (53.6–76.1)	109.2 (p < 0.0001)	90.8 (85.6–94.2)	22.3–97.1
All adults	68	15,401	33.3–100	92.0	91.8 (89.6–93.7)	1,087 (p < 0.0001)	93.8 (92.8–94.7)	71.0–100
All age-mixed	6	1,263	58.4–85.7	71.3	71.1 (60.7–80.6)	47.5 (p < 0.0001)	89.5 (79.7–94.5)	34.2–96.8
All studies	85	17,495	23.5–100	90.3	88.0 (85.3–90.5)	1,973.8 (p < 0.0001)	95.7 (95.2–96.2)	58.4–100

^a^Q: The Cochran’s Q statistic is a measure used here to assess the existence of heterogeneity in seroprevalence measures across studies.

^b^I^2^: A measure used here to assess the magnitude of between-study variation that is due to actual differences in seroprevalence across studies rather than chance.

^c^Prediction interval: A measure used here to estimate the distribution (the 95% interval) of true seroprevalence around the estimated pooled mean.

Abbreviations: CI = Confidence interval, HSV-1 = Herpes simplex virus type 1.

### Pooled mean seroprevalence estimates

Table 2 summarizes the results of the meta-analyses. In healthy general populations, the pooled mean HSV-1 seroprevalence was 65.2% (95% CI: 53.6–76.1%) for children, and 89.4% (95% CI: 87.3–91.4%) for adults. In adult clinical populations, the pooled mean HSV-1 seroprevalence was 95.3% (95% CI: 83.9–100%). Among other populations, the pooled mean HSV-1 seroprevalence was 95.2% (95% CI: 75.4–100%) in female sex workers, and 97.5% (95% CI: 93.5–99.7%) in mixed health-status populations.

By age group, the pooled mean HSV-1 seroprevalence was lowest at 60.5% (95% CI: 48.1–72.3%) in those aged <10 years, followed by 85.6% (95% CI: 80.5–90.1%) in those aged 10–19 years, 90.7% (95% CI: 84.7–95.5%) in those aged 20–29 years, and 94.3% (95% CI: 89.5–97.9%) in those aged ≥30 years. The sensitivity analyses using GLMM methods produced similar results (Supplementary Table S2).

Evidence of heterogeneity in seroprevalence was present in nearly all meta-analyses (p < 0.0001; Table 2). The I² measure indicated that most variation was attributed to true variability in seroprevalence across studies. The prediction intervals confirmed the considerable variation in seroprevalence across studies.

Forest plots of meta-analyses can be found in Supplementary Fig. S1.

### Predictors of seroprevalence and sources of between-study heterogeneity

Table 3 summarizes the results of the univariable and multivariable meta-regression models. In the univariable analyses, age bracket, age group, country’s income, population type, and sampling method had a p-value < 0.1 and were included in the multivariable analyses. Age bracket alone explained 44.3% of the variation in seroprevalence, followed by age group at 28.7%. Each of assay type, sample size, sex, year of data collection, and year of publication was not significantly associated with HSV-1 seroprevalence.

**Table 3:** Univariable and multivariable meta-regression analyses for herpes simplex virus type 1 (HSV-1) seroprevalence in the Middle East and North Africa.

		Studies	Samples	Univariable analysis			Multivariable analysis			
		Total N	Total n	*RR* (95% CI)	p-value	Variance explained adjusted R^2^ (%)	Model 1^a^		Model 2^b^	
							*ARR* (95% CI)	p-value	*ARR* (95% CI)	p-value
Age bracket	Children	11	831	1.0	—		1.0	—	—	—
	Adults	68	15,401	1.3 (1.2–1.5)	0.000		1.3 (1.2–1.5)	0.000	—	—
	Age-mixed	6	1,263	1.0 (0.9–1.2)	0.806	44.3	1.0 (0.9–1.2)	0.580	—	—
Age group	<10	9	639	1.0	—		—	—	1.0	—
	10–19	7	1,013	1.3 (1.1–1.6)	0.003		—	—	1.3 (1.1–1.6)	0.002
	20–29	8	980	1.4 (1.2–1.6)	0.000		—	—	1.4 (1.2–1.7)	0.000
	≥30	24	2,965	1.4 (1.2–1.6)	0.000		—	—	1.5 (1.3–1.7)	0.000
	Mixed	37	11,898	1.3 (1.2–1.5)	0.000	28.7	—	—	1.4 (1.2–1.6)	0.000
Assay type	ELISA	68	15,321	1.0	—		—	—	—	—
	EIA	2	155	1.0 (0.8–1.3)	0.915		—	—	—	—
	Nab	13	664	1.0 (0.9–1.1)	0.826		—	—	—	—
	IFA	1	1,186	1.1 (0.7–1.6)	0.687		—	—	—	—
	Western blot	1	169	1.1 (0.8–1.7)	0.434	0.0	—	—	—	—
Country’s income	LMIC	49	6,347	1.0	—		1.0	—	1.0	—
	UMIC	22	3,931	0.9 (0.8–1.0)	0.016		0.9 (0.8–1.0)	0.044	0.8 (0.8–1.0)	0.044
	HIC	12	7,030	0.9 (0.8–1.1)	0.440		0.9 (0.8–1.0)	0.076	0.9 (0.8–1.0)	0.101
	Mixed	2	187	1.0 (0.8–1.3)	0.822	3.3	1.0 (0.8–1.2)	0.847	1.0 (0.8–1.2)	0.848
Population type	Healthy general populations	64	13,776	1.0	—		1.0	—	1.0	—
	Clinical populations	11	530	1.0 (0.9–1.2)	0.355		1.0 (0.9–1.1)	0.967	1.0 (0.9–1.1)	0.987
	Other populations	10	3,189	1.1 (1.0–1.3)	0.064	4.6	1.0 (0.9–1.1)	0.839	1.0 (0.9–1.1)	0.706
Sample size^c^	<100	14	679	1.0	—		—	—	—	—
	≥100	71	16,816	1.0 (0.9–1.1)	0.712	0.0	—	—	—	—
Sampling method	Non-probability-based	81	14,704	1.0	—		1.0	—	1.0	—
	Probability based	4	2,791	0.8 (0.7–1.0)	0.070	9.3	0.9 (0.8–1.1)	0.621	0.9 (0.7–1.1)	0.251
Sex	Female	23	6,115	1.0	—		—	—	—	—
	Male	31	6,080	1.0 (0.9–1.1)	0.713		—	—	—	—
	Mixed	31	5,300	0.9 (0.8–1.0)	0.210	0.0	—	—	—	—
Year of data collection	85	17,495	1.0 (1.0–1.0)	0.993	0.0	—	—	—	—	—
Year of publication	85	17,495	1.0 (1.0–1.0)	0.911	0.0	—	—	—	—	—

^a^Variance explained by the final multivariable model 1 (adjusted *R*^2^) = 48.6%.

^b^Variance explained by the final multivariable model 2 (adjusted *R*^2^) = 40.2%.

^c^Sample size denotes the sample size of the study population found in the original publication.

Abbreviations: *ARR* = Adjusted relative risk, CI = Confidence interval, EIA = Enzyme immunoassay, ELISA = Enzyme-linked immunosorbent type-specific assay, HIC = High-income country, IFA = Immunofluorescence assay, LMIC = Lower-middle-income country, Nab = Neutralizing antibody assay, *RR* = Relative risk, UMIC = Upper-middle-income country.

To account for the fact that age bracket and age group both measure age, two final multivariable models were conducted. The first model included age bracket, country’s income, population type, and sampling method. This model explained 48.6% of seroprevalence variation. HSV-1 seroprevalence in adults was 1.3-fold (95% CI: 1.2–1.5) higher than in children. Seroprevalence in upper-middle-income countries and high-income countries was, in both, 0.9-fold (95% CI: 0.8–1.0) lower than in lower-middle-income countries. No association with population type and sampling method was found.

The second model included age group, assay type, country’s income, population type, and sampling method. The model explained 40.2% of seroprevalence variation, with similar results for country’s income, population type, and sampling method as in the first model. Compared to HSV-1 seroprevalence in those <10 years old, seroprevalence was 1.3-fold (95% CI: 1.1–1.6) higher in those 10–19 years old, 1.4-fold (95% CI: 1.2–1.7) higher in those 20–29 years old, and 1.5-fold (95% CI: 1.3–1.7) higher in those ≥30 years old.

### Quality assessment

Out of 32 records that included seroprevalence measures, only 15 were included in the systematic review with the remaining 17 being excluded due to potential issues in the validity of the diagnostic method, such as potential cross-reactivity with HSV-2 antibodies (Fig. 1). Of the studies included, 64.1% had high precision, 7.7% had low ROB for the sampling methodology domain, and 48.7% had low ROB for the response rate domain. These results, in context of the meta-regression models results, with different factors including sample size, sampling method, and assay type not being predictors of HSV-1 seroprevalence, suggest that overall the studies had reasonable quality.

The detailed quality assessment of included studies can be found in Supplementary Table S3.

## Discussion

HSV-1 epidemiology in MENA was investigated through a comprehensive systematic review and meta-analytics of existing evidence. HSV-1 seroprevalence was found at high level, suggesting considerable HSV-1-related morbidity that is yet to be quantified and tackled. Sixty-five percent of children and 90% of adults were found seropositive—seroprevalence increased rapidly with age at younger ages, and was consistent with most infections being acquired in childhood.

Remarkably, about half of the observed variation in seroprevalence was explained by factors set *a priori* and examined in this study. Age alone explained 44.3% of the variation. Despite improvements in socio-economic conditions and earlier speculation that seroprevalence levels may have been declining in MENA^47^, we did not find evidence for a declining trend over the last two decades. We also did not find evidence for variation in seroprevalence by sex, population type (healthy versus clinical), or study characteristics including assay type, sampling method, and sample size.

Though there was no evidence for recent declines in seroprevalence, youth had considerably lower HSV-1 seroprevalence than older subjects. As much as one-third of youth in MENA may be reaching sexual debut uninfected and thus potentially at risk of sexual acquisition, in context of recent evidence from Western countries and Asia reporting an increase in incidence of genital herpes attributed to HSV-1 rather than HSV-2^6,21–26^. We did not, nonetheless, identify any evidence for a potential role for HSV-1 sexual transmission in MENA. Despite the extensive search in multiple international, regional, and national databases, we failed to identify a single study that assessed the etiological role of HSV-1 in GUD or genital herpes in this region.

A comparison of the findings of the present study with that of a recent systematic review of HSV-1 in Asia^21^ demonstrates key insights about what may be general (or somewhat general) patterns in the global epidemiology of HSV-1 infection. Age in both systematic reviews was by far the strongest predictor of HSV-1 seroprevalence. Remarkably, in both MENA and Asia, seroprevalence in children was assessed at about 60%, and seroprevalence among adults was about 30% higher than that in children. Country’s income was also a predictor of seroprevalence with higher income associated with lower seroprevalence, attesting to an apparently global association between HSV-1 infection and socio-economic status^11,48^. However, the association of HSV-1 and socio-economic status differed between the two regions. In MENA, lower-middle-income countries had the highest seroprevalence, whereas in Asia, upper-middle-income countries had the highest seroprevalence. Possibly, the rapid modernization of Asia compared to MENA may contribute to explaining this difference.

In both MENA and Asia, sex, population type, assay type, sample size, and sampling method were not associated with HSV-1 seroprevalence. This suggests that HSV-1 is a truly general population infection that permeates all strata of society—there is no difficulty in sampling a representative sample provided the age distribution is representative. In both regions also, no evidence for a temporal trend in seroprevalence was identified despite the evidence for temporal declines in seroprevalence in Western countries^11,13–20^. Notably in MENA, half of the variation in seroprevalence (49%) was explained by the *a priori* considered factors, but only 26% of the variation was explained in Asia by the same considered factors^21^. The latter finding may relate to a higher population heterogeneity across countries in Asia than in MENA.

Our review and meta-analytics are limited by the quantity, quality, and representativeness of included studies. No data were identified for nine (mostly non-populous) of the 23 MENA countries, thereby potentially affecting the generalizability of the analyses to all of MENA. The number of seroprevalence measures varied from each study to another—only one (large) study, for example, contributed 29% of all stratified seroprevalence measures^47^. The majority of studies used convenience sampling (as opposed to probability-based sampling) of opportunistic populations such as blood donors or outpatients (Table 1). The latter, though, may not have been a limitation in context of the findings of the meta-regression analyses (Table 3).

Studies used different diagnostic methods, and such methods may differ in sensitivity and specificity^44,45^. Presence of HSV-2 antibodies may also affect diagnostic methods differentially, particularly the classic “relative-reactivity” methods such as IFA and Nab^49–51^. This limitation, however, may not have affected our results, as HSV-2 infection has a low seroprevalence in MENA^52^, and earlier work suggests minimal impact of this limitation on specifically HSV-1 seroprevalence (as opposed to HSV-2 seroprevalence)^49–51^. The meta-regression analyses found no variations in HSV-1 seroprevalence across assay types (Table 3).

There was extensive heterogeneity in HSV-1 seroprevalence measures, but half of this heterogeneity was subsequently explained by only two factors, age and country’s income (Table 3). Lastly, no study of HSV-1 viral detection in GUD or in genital herpes in MENA was identified, thus limiting our ability to assess the epidemiological role of HSV-1 sexual transmission. In spite of these limitations, our study is the first to draw a comprehensive synthesis and analytics of HSV-1 seroprevalence for the MENA region, and to highlight opportunities for related research and public health response.

## Conclusions

HSV-1 seroprevalence in MENA indicated that 65% of children and 90% of adults had been exposed to this infection, by inference, most often during childhood. Age and country’s income were the strongest predictors of HSV-1 seroprevalence and explained half of seroprevalence variation. No evidence was found for a temporal trend in seroprevalence over the last two decades despite improvements in socio-economic conditions. With no identified study of HSV-1 viral detection in GUD or in genital herpes, the role of HSV-1 sexual transmission in MENA remains unknown. This lack of data calls for at least basic or opportunistic GUD/genital herpes etiological surveillance. The totality of the findings highlights the timeliness of accelerating HSV-1 vaccine development to control one of the most endemic infections worldwide.

## Supplementary information

Supplementary Material
