# Supplementary material for: Herpes simplex virus type 1 epidemiology in the Middle East and North Africa: systematic review, meta-analyses, and meta-regressions
Source: Sci Rep. 2019 Feb 4;9:1136. doi: 10.1038/s41598-018-37833-8 (PMC6362060; doi:10.1038/s41598-018-37833-8)
Supplement: Supplementary file 1 — Supplementary Material [file 41598_2018_37833_MOESM1_ESM.docx]

**SuppLEMENTARY MATERIAL**

**Herpes simplex virus type 1 epidemiology in the Middle East and North Africa: systematic review, meta-analyses, and meta-regressions**

Sonia Chaabane^1^, Manale Harfouche^1^, Hiam Chemaitelly^1^, Guido Schwarzer^2^, and Laith J. Abu-Raddad^1,3,4*^

^1^*Infectious Disease Epidemiology Group, Weill Cornell Medicine-Qatar, Cornell University, Qatar Foundation - Education City, Doha, Qatar*

*^2^Institute of Medical Biometry and Statistics, Faculty of Medicine and Medical Center - University of Freiburg, Germany*

*^3^Department of Healthcare Policy & Research, Weill Cornell Medicine, Cornell University, New York, USA*

*^4^College of Health and Life Sciences, Hamad bin Khalifa University, Doha, Qatar*

***Reprints or correspondence:** Professor Laith J. Abu-Raddad, PhD, Infectious Disease Epidemiology Group, Weill Cornell Medicine–Qatar, Qatar Foundation - Education City, P.O. Box 24144, Doha, Qatar. Telephone: +(974) 4492-8321. Fax: +(974) 4492-8333. E-mail: [lja2002@qatar-med.cornell.edu](mailto:lja2002@qatar-med.cornell.edu).

**Supplementary Table S1.** Preferred Reporting Items for Systematic Reviews and Meta-analyses (PRISMA) checklist^1^.

| **Section/topic** | **#** | **Checklist item** | **Reported in main text on** |
| --- | --- | --- | --- |
| **TITLE: The epidemiology of herpes simplex virus type 1 seroprevalence in the Middle-East and North Africa: a systematic review and meta-analysis** | | |  |
| Title | 1 | Identify the report as a systematic review, meta-analysis, or both. | p. 1 |
| **ABSTRACT** | | |  |
| Structured summary | 2 | Provide a structured summary including, as applicable: background; objectives; data sources; study eligibility criteria, participants, and interventions; study appraisal and synthesis methods; results; limitations; conclusions and implications of key findings; systematic review registration number. | p. 2 |
| **INTRODUCTION** | | |  |
| Rationale | 3 | Describe the rationale for the review in the context of what is already known. | p. 3-4 |
| Objectives | 4 | Provide an explicit statement of questions being addressed with reference to participants, interventions, comparisons, outcomes, and study design (PICOS). | p. 3-4 |
| **METHODS** | | |  |
| Protocol and registration | 5 | Indicate if a review protocol exists, if and where it can be accessed (e.g., Web address), and, if available, provide registration information including registration number. | NA |
| Eligibility criteria | 6 | Specify study characteristics (e.g., PICOS, length of follow-up) and report characteristics (e.g., years considered, language, publication status) used as criteria for eligibility, giving rationale. | p. 5 |
| Information sources | 7 | Describe all information sources (e.g., databases with dates of coverage, contact with study authors to identify additional studies) in the search and date last searched. | p. 4 |
| Search | 8 | Present full electronic search strategy for at least one database, including any limits used, such that it could be repeated. | Supplementary Box S1 |
| Study selection | 9 | State the process for selecting studies (i.e., screening, eligibility, included in systematic review, and, if applicable, included in the meta-analysis). | p. 5 |
| Data collection process | 10 | Describe method of data extraction from reports (e.g., piloted forms, independently, in duplicate) and any processes for obtaining and confirming data from investigators. | p. 6 |
| Data items | 11 | List and define all variables for which data were sought (e.g., PICOS, funding sources) and any assumptions and simplifications made. | p. 6 |
| Risk of bias in individual studies | 12 | Describe methods used for assessing risk of bias of individual studies (including specification of whether this was done at the study or outcome level), and how this information is to be used in any data synthesis. | p. 8-9 |
| Summary measures | 13 | State the principal summary measures (e.g., risk ratio, difference in means). | p. 6-8 |
| Synthesis of results | 14 | Describe the methods of handling data and combining results of studies, if done, including measures of consistency (e.g., I^2^) for each meta-analysis. | p. 6-8 |
| Risk of bias across studies | 15 | Specify any assessment of risk of bias that may affect the cumulative evidence (e.g., publication bias, selective reporting within studies). | p. 8 |
| Additional analyses | 16 | Describe methods of additional analyses (e.g., sensitivity or subgroup analyses, meta-regression), if done, indicating which were pre-specified. | p. 7-8 |
| **RESULTS** | | |  |
| Study selection | 17 | Give numbers of studies screened, assessed for eligibility, and included in the review, with reasons for exclusions at each stage, ideally with a flow diagram. | p. 9; Figure 1 |
| Study characteristics | 18 | For each study, present characteristics for which data were extracted (e.g., study size, PICOS, follow-up period) and provide the citations. | p. 10; and Table 1 |
| Risk of bias within studies | 19 | Present data on risk of bias of each study and, if available, any outcome level assessment (see item 12). | p. 12; Supplementary Table S2 |
| Results of individual studies | 20 | For all outcomes considered (benefits or harms), present, for each study: (a) simple summary data for each intervention group (b) effect estimates and confidence intervals, ideally with a forest plot. | p. 9-11; Table 1; Supplementary Figure S1 |
| Synthesis of results | 21 | Present results of each meta-analysis done, including confidence intervals and measures of consistency. | p.10-11; Table 2; Supplementary Figure S1 |
| Risk of bias across studies | 22 | Present results of any assessment of risk of bias across studies (see Item 15). | p. 12; Supplementary Table S2 |
| Additional analysis | 23 | Give results of additional analyses, if done (e.g., sensitivity or subgroup analyses, meta-regression [see Item 16]). | p. 11 and Table 3 |
| **DISCUSSION** | | |  |
| Summary of evidence | 24 | Summarize the main findings including the strength of evidence for each main outcome; consider their relevance to key groups (e.g., healthcare providers, users, and policy makers). | p. 12 |
| Limitations | 25 | Discuss limitations at study and outcome level (e.g., risk of bias), and at review-level (e.g., incomplete retrieval of identified research, reporting bias). | p. 14 |
| Conclusions | 26 | Provide a general interpretation of the results in the context of other evidence, and implications for future research. | p. 12-14 |
| **FUNDING** | | |  |
| Funding | 27 | Describe sources of funding for the systematic review and other support (e.g., supply of data); role of funders for the systematic review. | p. 21 |

Abbreviations: NA = Not applicable, p = page(s).

**Supplementary Box S1.** Data sources and search criteria for systematically reviewing herpes simplex virus type 1 (HSV-1) studies in the Middle East and North Africa.

**PubMed (last searched: October 8, 2017)**

("Herpesvirus 1, Human"[Mesh] OR "Simplexvirus"[Mesh] OR ("Herpes Simplex"[Mesh]) OR "HSV type-1"[Text] OR "HSV type 1"[Text] OR "HSV1"[Text] OR "HSV-1"[Text] OR "HSV 1"[Text] OR "Human herpes virus"[Text] OR "Herpes simplex virus type 1 "[Text] OR "Herpes simplex virus type-1"[Text] OR "herpes simplex virus 1"[Text] OR "herpes simplex virus-1"[Text] OR "herpes simplex type 1"[Text] OR "herpes simplex type-1"[Text] OR "herpes simplex 1"[Text] OR "herpes simplex-1"[Text] OR "Herpesvirus type 1"[Text] OR "Herpesvirus type-1"[Text] OR "Herpesvirus 1"[Text] OR "Herpesvirus-1"[Text] OR "Herpes virus type 1"[Text] OR "Herpes virus type-1"[Text] OR "Herpes virus 1"[Text] OR "Herpes virus-1"[Text] OR "genital herpes"[Text] OR "Herpes Genitalis"[Text] or "Herpes Genitalis"[Text] OR "Stomatitis Herpetic"[Text] OR "Herpes Labialis"[ Text]) AND ("Middle East"[Mesh] OR "Islam"[Mesh] OR "Arabs"[Mesh] OR "Arab World"[Mesh] OR "Africa, Northern"[Mesh] OR "Sudan"[Mesh] OR "Somalia"[Mesh] OR "Djibouti"[Mesh] OR "Pakistan"[Mesh] OR "Middle East"[Text] OR "Middle-East"[Text] OR "North Africa"[Text] OR "North-Africa"[Text] OR "EMRO"[Text] OR "Eastern Mediterranean"[Text] OR "Arab"[Text] OR "Arabs"[Text] OR "Arab World"[Text] OR "Islam"[Text] OR "Afghanistan"[Text] OR "Algeria"[Text] OR "Bahrain"[Text] OR "Djibouti"[Text] OR "Egypt"[Text] OR "Jordan"[Text] OR "Kuwait"[Text] OR "Lebanon"[Text] OR "Libya"[Text] OR "Iran"[Text] OR "Iraq"[Text] OR "Morocco"[Text] OR "Oman"[Text] OR "Pakistan"[Text] OR "Qatar"[Text] OR "Saudi Arabia"[Text] OR "Somalia"[Text] OR "Sudan"[Text] OR "Syria"[Text] OR "Tunisia"[Text] OR "United Arab Emirates"[Text] OR "Dubai"[Text] OR "Abu Dhabi"[Text] OR "Abu-Dhabi"[Text] OR "Sharjah"[Text] OR "West Bank"[Text] OR "Ghaza"[Text] OR "Palestine"[Text] OR "Yemen"[Text])

**Embase (last searched: October 8, 2017)**

(exp Herpes simplex virus or exp herpes simplex OR (Herpes simplex or Herpes simplex virus or HSV type-1 or HSV type 1 or HSV1 or HSV-1 or HSV 1 or human herpes virus or Herpes simplex virus type 1 or Herpes simplex virus type-1 or herpes simplex virus 1 or herpes simplex virus-1 or herpes simplex type 1 or herpes simplex type-1 or herpes simplex 1 or herpes simplex-1 or Herpesvirus type 1 or Herpesvirus type-1 or Herpesvirus 1 or Herpesvirus-1 or Herpes virus type 1 or Herpes virus type-1 or Herpes virus 1 or Herpes virus-1 or genital herpes or Herpes Genitalis or herpes labialis or herpetic stomatitis).mp. AND (exp Middle East/ or exp North Africa/ or exp Arab/ or exp Afghanistan/ or exp Djibouti/ or exp Pakistan/ or exp Somalia/ or exp Sudan/ or Middle East.mp. or North Africa.mp. or EMRO.mp. or Eastern Mediterranean.mp. or Arab.mp. or Arabs.mp. or Arab World.mp. or Islam.mp. or Afghanistan.mp. or Algeria.mp. or Bahrain.mp. or Djibouti.mp. or Egypt.mp. or Jordan.mp. or Kuwait.mp. or Lebanon.mp. or Libya.mp. or Iran.mp. or Iraq.mp. or Morocco.mp. or Oman.mp. or Pakistan.mp. or Qatar.mp. or Saudi Arabia.mp. or Somalia.mp. or Sudan.mp. or Syria.mp. or Tunisia.mp. or United Arab Emirates.mp. or Dubai.mp. or Abu Dhabi.mp. or Sharjah.mp. or West Bank.mp. or Ghaza.mp. or Palestine.mp. or Yemen.mp.)

**National and regional databases^a^**

1. **Index Medicus for the Eastern Mediterranean Region (last searched: December 1, 2017)**

Herpes or Herpes simplex or HSV

1. **Iraqi Academic Scientific Journals Database (last searched: December 1, 2017)**

Herpes or Herpes simplex

1. **Scientific Information Database of Iran (last searched: December 1, 2017)**

Herpes or Herpes simplex or Herpesvirus

1. **PakMediNet of Pakistan (last searched: December 1, 2017)**

Herpes

**^A^**

^a^ The actual search terms differed from one national/regional database to another depending on the terms indexed or used by that database.

**Table 2**. Comparison of the pooled mean estimates for herpes simplex virus type 1 (HSV-1) seroprevalence in different populations in the Middle East and North Africa using the Freeman-Tukey type arcsine square-root transformation versus the generalized linear mixed models (GLMM).

| **Population type** | **Studies** | **Samples** | **Pooled mean HSV-1 seroprevalence using the Freeman-Tukey type arcsine square-root transformation** | | | | **Pooled mean HSV-1 seroprevalence using the generalized linear mixed models** | |  |
| --- | --- | --- | --- | --- | --- | --- | --- | --- | --- |
|  | **Total**  **N** | **Total**  **N** | **Random-effects models**  **(95% CI)** | **Fixed-effects models (95% CI)** | | **Random-effects models**  **(95% CI)** | | **Fixed-effects models (95% CI)** |  |
| **Healthy general populations** | | | | | | | | |  |
| Children | 11 | 831 | 65.2 (53.6-76.1) | | 70.1 (66.9-73.2) | | 66.0 (53.6-76.6) | 68.9 (65.7-72.0) |  |
| Adults | 49 | 11,754 | 89.4 (87.3-91.4) | | 89.0 (88.4-89.6) | | 90.9 (87.6-93.4) | 87.9 (87.3-88.5) |  |
| Age-mixed | 4 | 1,191 | 71.1 (58.5-82.3) | | 63.7 (60.9-66.4) | | 71.4 (60.0-80.7) | 63.3 (60.5-66.0) |  |
| All healthy general populations | 64 | 13,776 | 85.3 (82.3-87.9) | | 86.3 (85.7-86.9) | | 87.4 (83.4-90.5) | 84.6 (84.0-85.2) |  |
| **Clinical populations** | | | | | | | | |  |
| Children | - | - | - | | - | | - | - |  |
| Adults | 9 | 458 | 95.3 (83.9-100) | | 93.5 (90.8-95.8) | | 97.5 (89.9-99.5) | 88.2 (84.9-90.8) |  |
| Age-mixed | 2 | 72 | - | | - | | - | - |  |
| All clinical populations | 11 | 530 | 92.3 (80.3-99.4) | | 90.8 (88.0-93.3) | | 95.8 (85.9-98.9) | 85.3 (82.0-88.0) |  |
| **Other populations** | | | | | | | | |  |
| Female sex workers | 4 | 226 | 95.2 (75.4-100) | | 93.2 (89.4-96.3) | | 98.9 (70.8-100) | 88.0 (83.1-91.7) |  |
| Healthy/clinical adult populations | 6 | 2,963 | 97.5 (93.5-99.7) | | 97.5 (97.0-98.1) | | 98.7 (92.1-99.9) | 96.3 (95.5-96.9) |  |
| **Age group** | | | | | | | | |  |
| <10 years | 9 | 639 | 60.5 (48.1-72.3) | | 64.7 (60.9-68.5) | | 61.0 (47.9-72.7) | 64.0 (60.2-67.6) |  |
| 10-19 years | 7 | 1,013 | 85.6 (80.5-90.1) | | 88.5 (86.4-90.5) | | 85.9 (81.5-89.5) | 87.8 (85.7-89.7) |  |
| 20-29 years | 8 | 980 | 90.7 (84.7-95.5) | | 91.8 (89.9-93.5) | | 91.4 (83.6-95.6) | 90.6 (88.6-92.3) |  |
| ≥30 years | 24 | 2,965 | 94.3 (89.5-97.9) | | 97.7 (97.1-98.3) | | 97.1 (93.1-98.8) | 94.3 (93.4-95.1) |  |
| All children | 11 | 831 | 65.2 (53.6-76.1) | | 70.1 (66.9-73.2) | | 66.0 (53.6-76.6) | 68.9 (65.7-72.0) |  |
| All adults | 68 | 15,401 | 91.8 (89.6-93.7) | | 91.5 (91.0-91.9) | | 94.0 (91.4-95.9) | 89.5 (89.0-90.0) |  |
| All age-mixed | 6 | 1,263 | 71.1 (60.7-80.6) | | 64.0 (61.3-66.7) | | 71.3 (61.3-79.7) | 63.5 (60.8-66.1) |  |
| **All studies** | **85** | **17,495** | **88.0 (85.3-90.5)** | | **89.2 (88.7-89.6)** | | **91.3 (87.9-93.8)** | **86.7 (86.2-87.2)** |  |

Abbreviations: HSV-1 = Herpes simplex type 1, CI = Confidence interval.

**Supplementary Figure S1.** Forest plots presenting the outcomes of the pooled mean herpes simplex virus type 1 (HSV-1) seroprevalence in different populations in the Middle East and North Africa.

1. **All children populations**

**
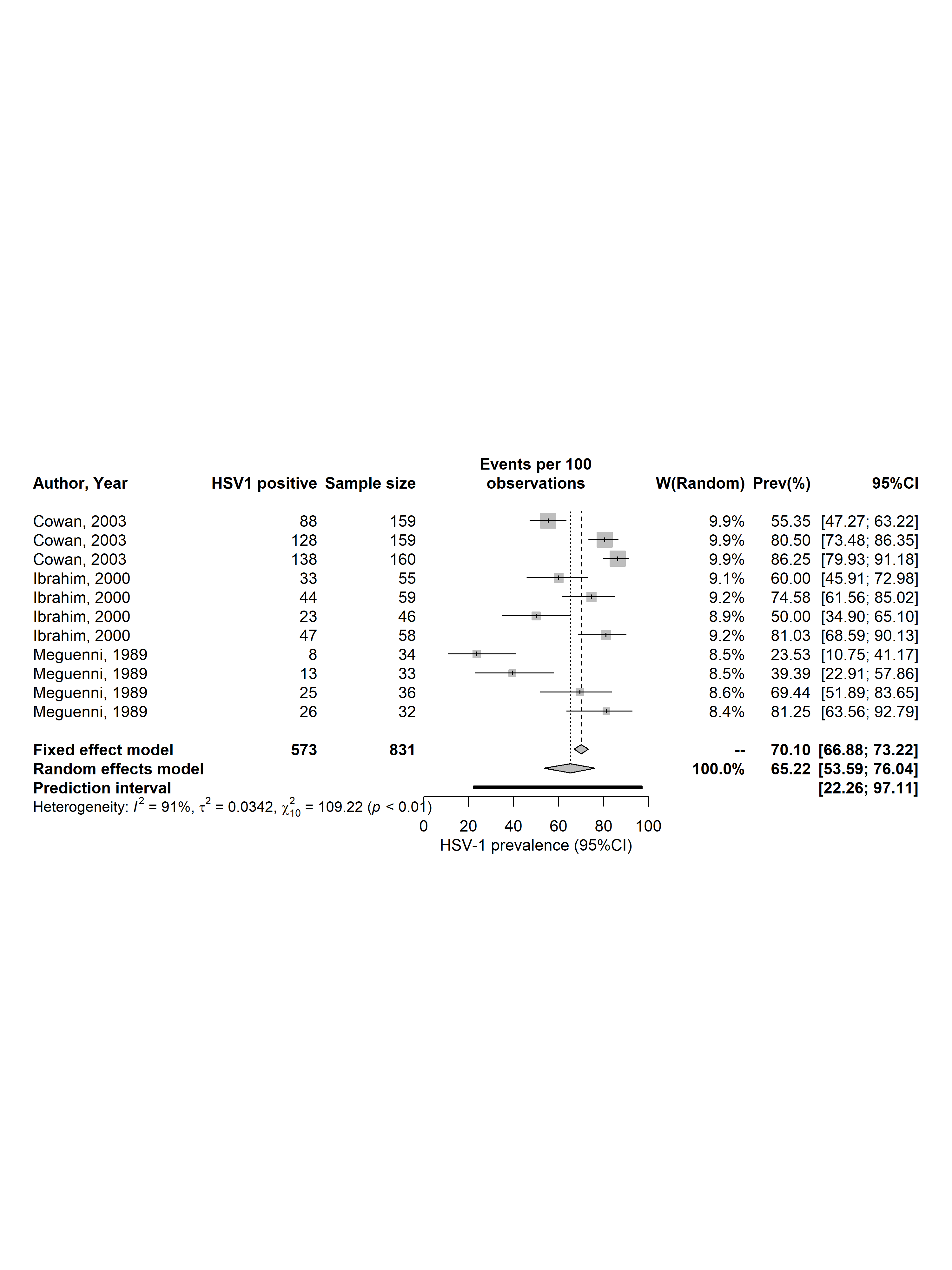
**

1. **All adult populations**

**
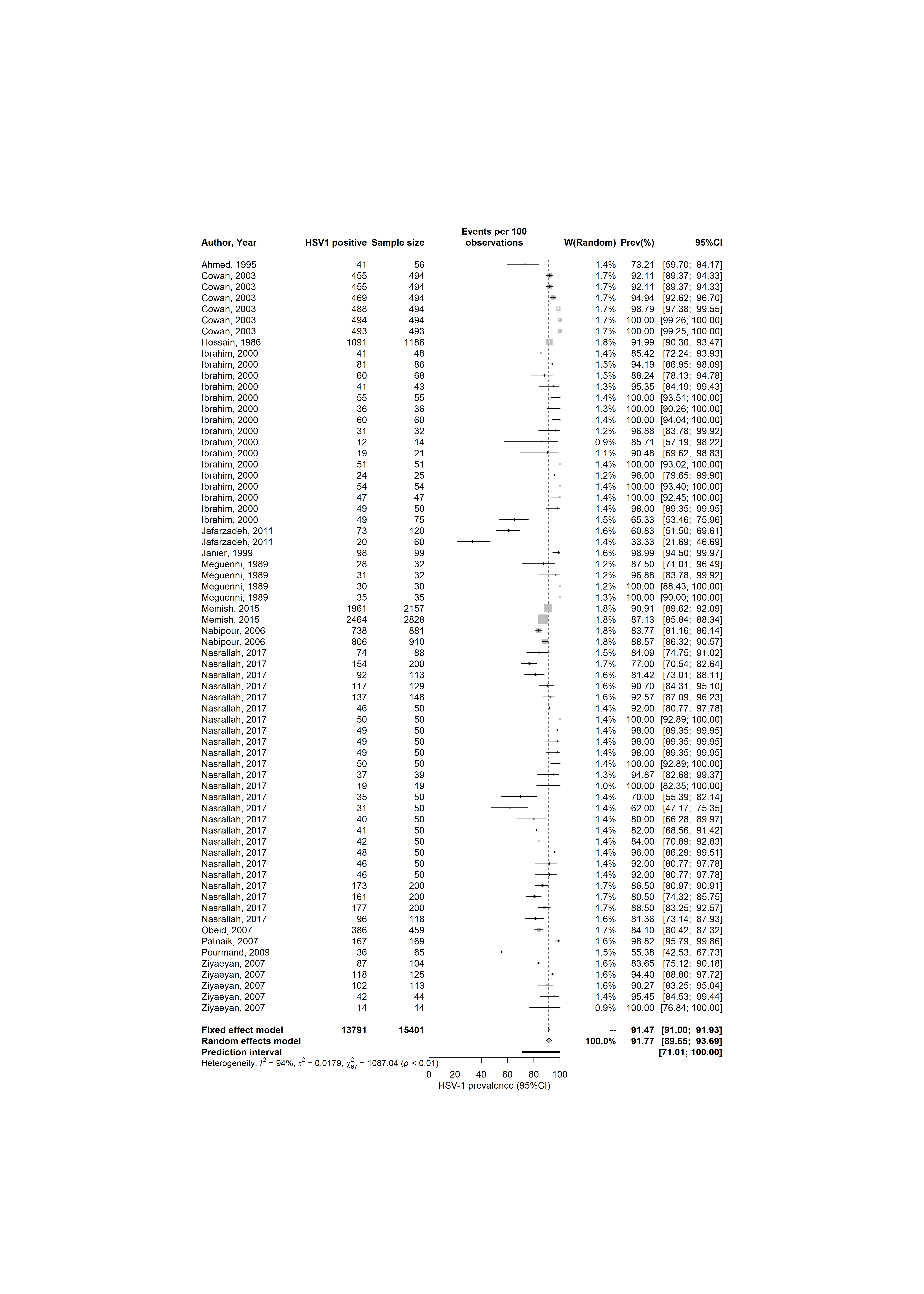
**

1. **All age-mixed populations**

**
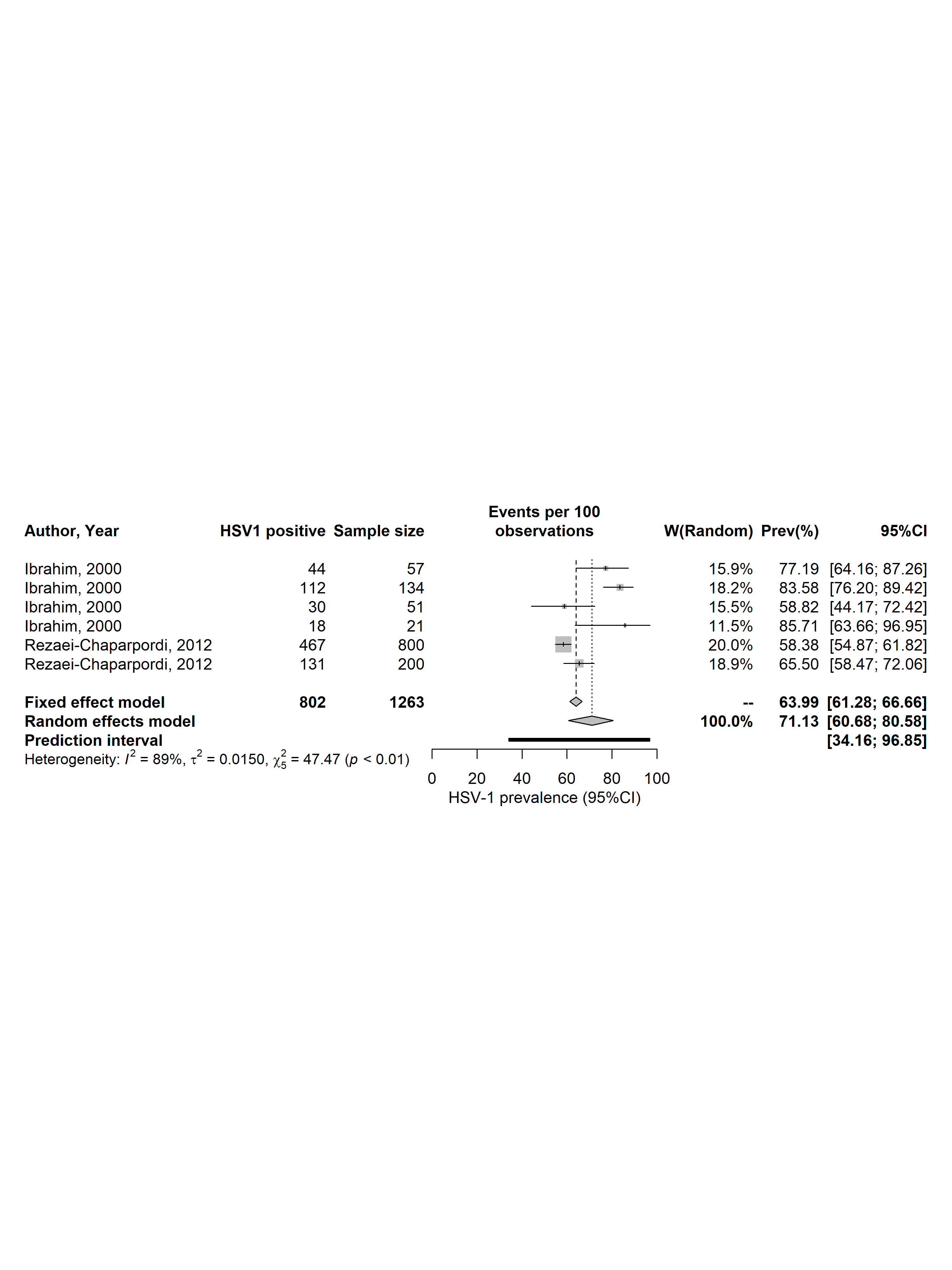
**

**Supplementary Table S3.** Summary of the precision and risk of bias (ROB) assessment of the studies reporting herpes simplex virus type 1 (HSV-1) seroprevalence in the Middle East and North Africa.

| **Quality assessment** | **HSV-1 seroprevalence measures** | |
| --- | --- | --- |
|  | **Number of studies** | **%** |
| **Precisions of prevalence measures^a^** | | |
| Low precision | 14 | 35.9 |
| High precision | 25 | 64.1 |
| **Risk of bias quality domain^b^** | | |
| **Sampling methodology** | | |
| Low risk of bias | 3 | 7.7 |
| High risk of bias | 36 | 92.3 |
| **Response rate** | | |
| Low risk of bias | 19 | 48.7 |
| High risk of bias | 2 | 5.1 |
| Unclear risk of bias | 18 | 46.1 |
| **Summary of the risk of bias assessment** | | |
| **Low risk of bias** |  |  |
| In at least one quality domain | 20 | 51.3 |
| In both quality domains | 2 | 5.1 |
| **High risk of bias** |  |  |
| In at least one quality domain | 36 | 92.3 |
| In both quality domains | 3 | 7.6 |
| **Total seroprevalence studies where risk of bias assessment was possible** | **39** | **100** |

^a^ Precision was assessed based on the overall sample size (not the strata) of the study as reported in the record/publication.

^b^ Risk of bias was assessed based on the overall sample size (not the strata) of the study as reported in the record/publication.

Abbreviation: HSV-1 = Herpes simplex virus type 1.

**References**

1 Moher, D., Liberati, A., Tetzlaff, J., Altman, D. G. & Group, P. Preferred reporting items for systematic reviews and meta-analyses: the PRISMA statement. *PLoS med* **6**, e1000097 (2009).
